# Nosocomial outbreak of *Ralstonia mannitolilytica* associated with prefilled saline flush syringes distributed in Europe, Germany, March to September 2026

**DOI:** 10.2807/1560-7917.ES.2026.31.40.2600819

**Published:** 2026-10-08

**Authors:** Sofia Burdi, Lukas Herrmann, Sophia N. Koerdt, Silver A. Wolf, Sandra Appelt, Mirco Sandfort, Barbara Maier, Sandra Reuter, Martin J. Hug, Nicholas Jakob Böhm, Gizem Karadag, Georg Berndt, Achim Dörre, Vera Schwierzeck, Linda Behrends, Pascal Kreuzer, Julia Kuhn, Lukas Murajda, Saskia Roßbach, Susanne Kolbe-Busch, Cornelia Seidel, Patrick Jung, Grettel Chanto Chacón, Irina Zuschneid, Somayyeh Sedaghatjoo, Maximilian Driller Povill, Holger C. Scholz, Alexander Mellmann, Tim Eckmanns, Josef Zündorf, Michael Hogardt, Sebastian Haller

**Affiliations:** 1 Department of Infectious Disease Epidemiology, Robert Koch Institute, Berlin, Germany; 2 Postgraduate Training for Applied Epidemiology (PAE), Department for Infectious Disease Epidemiology, Robert Koch Institute, Berlin, Germany; 3 European Programme for Intervention Epidemiology Training (EPIET), European Centre for Disease Prevention and Control, Stockholm, Sweden; 4 Federal Institute for Drugs and Medical Devices (BfArM), Bonn, Germany; 5 Genome Competence Centre (MF 1), Robert Koch Institute, Berlin, Germany; 6 Centre for Biological Threats and Special Pathogens (ZBS 2), Robert Koch Institute, Berlin, Germany; 7 Institute for Infection Control and Prevention, Medical Center – University of Freiburg, Freiburg, Germany; 8 Pharmacy, Medical Center - University of Freiburg, Freiburg, Germany; 9 Institute of Hygiene and Environmental Medicine, Charité - Universitätsmedizin Berlin, Berlin, Germany; 10 National Reference Centre for Surveillance of Nosocomial Infections, Berlin, Germany; 11 Pharmacy, Charité-Universitätsmedizin Berlin, Berlin, Germany; 12 Institute for Hygiene, University Hospital Münster, Münster, Germany; 13 Health Authority Düsseldorf, Düsseldorf, Germany; 14 Department of Health Protection, Infection control and Epidemiology State Health Office, Ministry of Social Affairs, Labour and Health Baden-Wuerttemberg, Stuttgart, Germany; 15 Health Authority Berlin-Mitte, Berlin, Germany; 16 Infection Prevention and Control Unit, University Hospital Düsseldorf, Düsseldorf, Germany; 17 Institute of Hygiene, Hospital Epidemiology and Environmental Health, University of Leipzig Medical Center, Leipzig, Germany; 18 Städtisches Klinikum Görlitz, Görlitz, Germany; 19 Städtisches Klinikum Karlsruhe, Karlsruhe, Germany; 20 National Reference Centre for Bacteriology, Inciensa, Tres Ríos, Costa Rica; 21 Health Authority Charlottenburg-Wilmersdorf, Berlin, Germany; 22 Institute for Medical Microbiology and Infection Control, University Hospital Frankfurt, Goethe University Frankfurt, Frankfurt am Main, Germany; 23 German National Consiliary Laboratory on Cystic Fibrosis Bacteriology, Frankfurt am Main, Germany

**Keywords:** Ralstonia, Disease Outbreaks, Infection Control, Healthcare-Associated Infections, Whole Genome Sequencing

## Abstract

A nosocomial outbreak of *Ralstonia mannitolilytica* bloodstream infections in Germany from March to September 2026 included 16 cases (≤ 6 allelic differences using core genome multilocus sequence typing) across seven hospitals. Epidemiological investigations supported by a large language model, identified prefilled saline flush syringes of one manufacturer as common exposure across all cases. The manufacturer initiated a temporary quarantine and suspension of use of this product. Because it was distributed to other countries, cases may have occurred elsewhere, prompting this report.

We report a multicentre nosocomial outbreak of *Ralstonia mannitolilytica* in Germany, from March 2026 to September 2026, which was epidemiologically linked to internationally distributed prefilled saline flush syringes. In total, 16 confirmed cases with *R. mannitolilytica* were identified across seven hospitals in four German federal states. Aiming to alert international readers to the possibility of related cases and similar events elsewhere, we describe the outbreak investigation and findings.

## Outbreak identification

In April 2026, two university hospitals in Germany independently reported several bloodstream infections with *R. mannitolilytica* without obvious epidemiological links. The Antibiotic Resistance Surveillance (ARS) database, which comprises all routine clinical microbiological test results from a country-wide voluntary laboratory network covering about 50% of all acute care German hospitals [1] was reviewed. This revealed an apparent increase of *R. mannitolilytica* detection in blood cultures (usually 0–1 annually, 7 from January to April 2026), prompting an outbreak investigation.

## Molecular and epidemiological investigation

A confirmed case was defined as an individual with *R. mannitolilytica* detected in a blood culture since 1 January 2026 in Germany, with an isolate sequence that could be linked to the genetic outbreak cluster within an allele distance of ≤ 6 alleles based on core genome multilocus sequence typing (cgMLST).

For case finding, the Robert Koch Institute (RKI), which is Germany’s national public health institute, introduced within Germany’s nationwide mandatory surveillance [2], a notification mandate for *R. mannitolilytica* detection between 1 January and 31 August 2026 in any specimen. We monitored mandatory notifications, as well as ARS, for any *R. mannitolilytica* identifications. In the event of a detection, we contacted the responsible laboratory to request isolates or sequences and respective records of the affected patients. We conducted Illumina whole genome-sequencing and a cgMLST-based cluster analysis and compared the outbreak isolates to those of other outbreaks, including an ongoing outbreak cluster in Costa Rica identified through ProMED-Mail search (http://www.promedmail.org), and to all available *R. mannitolilytica* isolates from any specimen collected since November 2025 in Germany.

We informed the regional and local public health authorities through our weekly epidemiological teleconference EpiLag, the microbiological laboratories and hospital hygiene departments through the German Society for Hygiene and Microbiology (DGHM), the European Centre for Disease Prevention and Control (ECDC), public health institutions through the EpiPulse platform, and the German Federal Institute for Drugs and Medical Devices (BfArM) about the outbreak.

In total, we detected 16 confirmed cases in Germany. Figure 1 shows confirmed cases by the calendar week of first detection and by the hospital in which they were hospitalised.

**Figure 1:**
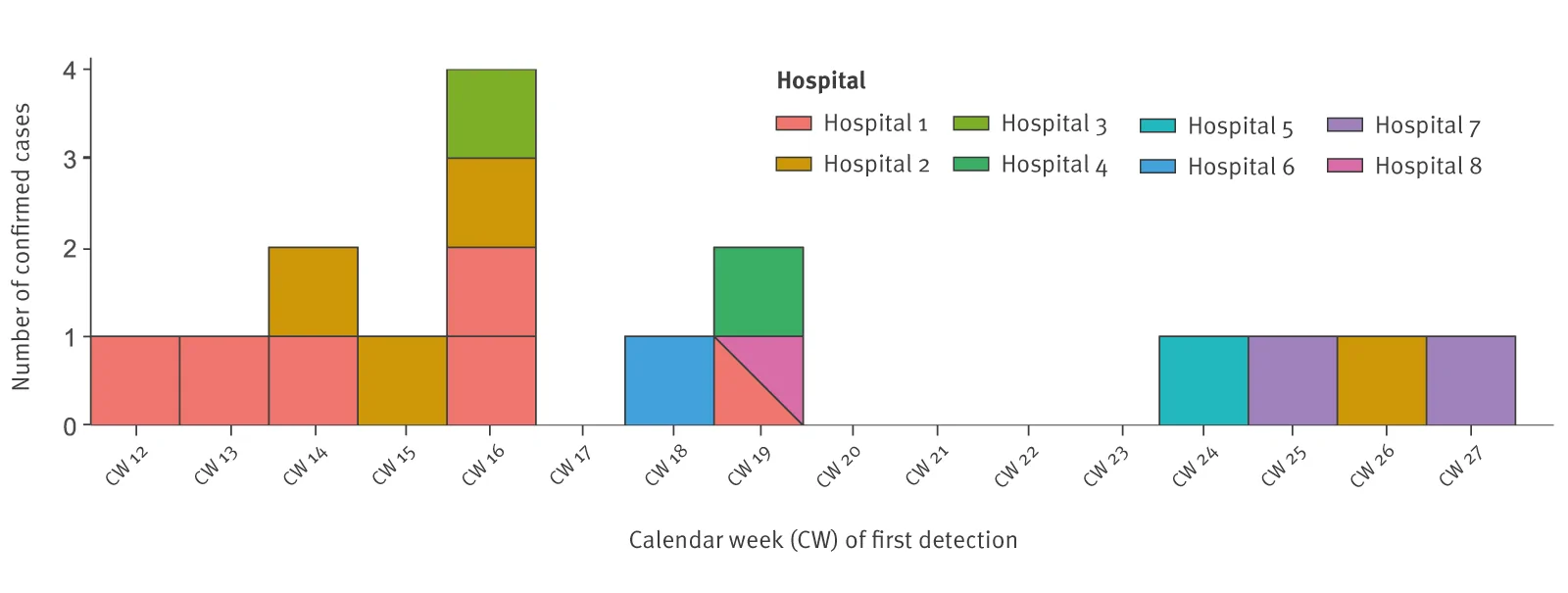
Confirmed cases of *Ralstonia mannitolilytica* by calendar week of first positive sampling and by hospital in which they were hospitalised, Germany, March 2026–September 2026 (n = 16 cases)^a^

^a^ Data up to 30 September 2026.

One case was detected in Hospital 8 but exposure most likely occurred in Hospital 1, where the case was treated 2 weeks before.

While the 16 cases were detected in eight hospitals, the only case in Hospital 8 had likely been infected in Hospital 1 where this case was prior treated. The 16 confirmed cases thus likely occurred in seven hospitals across four federal states in Germany (excluding Hospital 8). All confirmed cases had received intravenous medications. In 11 cases, the blood culture was obtained because of clinical signs of systemic infection; in the rest of the cases blood cultures were obtained as part of clinical routine irrespective of signs of infection. The Table summarises the demographic and clinical characteristics of the cases. The hospital and ward histories revealed no overlapping stays. Together with the supra-regional occurrence of cases and the absence of epidemiological links such as previous travels, a point source appeared likely.

**Table:** Characteristics of confirmed *Ralstonia mannitolilytica* cases in Germany, March 2026–September 2026 (n = 16 cases)^a^

Case characteristics	Confirmed cases (n = 16)
**Age**	**Median (minimum and maximum range)**
Age in years	62 (1–91)
**Sex**	**Number**
Female	5
Male	11
**Underlying disease**	**Number**
Haematologic/oncologic malignancy	11
Heart failure	2
Liver failure	2
Trauma	1
**Sepsis**	**Number**
Yes	3
No	10
Unknown	3
**Deceased**	**Number**
Yes^b^	4
No	11
Unknown	1

^a^ Data up to 30 September 2026.

^b ^One death attributed to the *R. mannitolilytica* infection.

We identified 18 bloodstream infections caused by *R. mannitolilytica*. Of these, 16 isolates were available for sequencing, all belonging to one distinct cluster (Figure 2). The two isolates that could not be sequenced originated from two different hospitals, none of which was associated with a confirmed case. The two specimens were collected in calendar weeks 9, and 17, respectively. Among *R. mannitolilytica* found in any specimen collected since November 2025 in Germany, isolates available from detections outside of blood culture did not cluster genetically. Moreover, one blood culture isolate from 2025 did not cluster either. These three isolates were thus excluded from the investigation.

**Figure 2:**
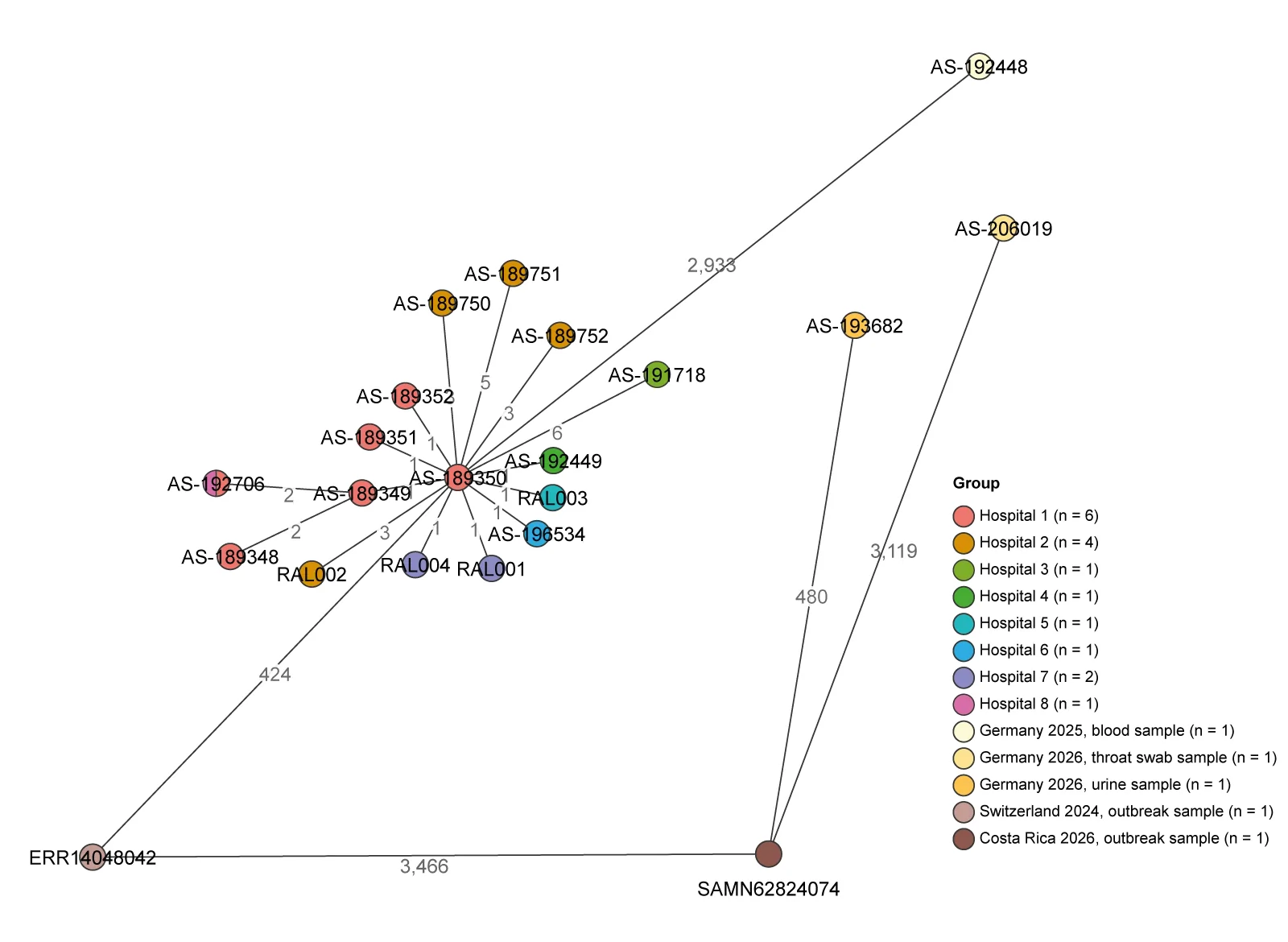
Minimum spanning tree of *Ralstonia mannitolilytica* sequences after cgMLST, including sequences from confirmed cases of the outbreak in Germany, March 2026–September 2026 (n = 16 confirmed cases)^a^

cgMLST: core genome-multilocus sequence typing.

^a^ The tree is based on 21 nt sequences, and these include the sequences of 16 confirmed cases of a *Ralstonia mannitolilytica* outbreak in Germany. Data are up to 30 September 2026.

One of publicly available sequences from a recent outbreak in Switzerland [10] was used for the minimum spanning tree, as well as one sequence from an ongoing outbreak in Costa Rica and sequences of available isolates from Germany including one from a blood culture in 2025, one from a throat swab in 2026, and one from a urine sample in 2026. The cgMLST scheme comprises 3,724 loci; scheme creation and subsequent cgMLST calling was performed using chewBBACA (v3.5.4); the tree was visualised using GrapeTree with the MSTree2 algorithm. Edge lengths are visualised logarithmically.

## Investigation of common exposures

The investigation of common exposures among confirmed cases comprised two sequential steps: (i) hypothesis generation leading to identification of suspected exposures, and (ii) targeted assessment of suspected exposures using a questionnaire administered to all hospitals with confirmed cases at the time. We focused our analysis on substances and products administered intravenously within 20 days before *R. mannitolilytica* detection. For hypothesis generation, we systematically compared patient-level exposures individually documented in the patients’ files. As not all exposures may be documented at the individual case level, ward-specific supply data from hospital pharmacies and purchasing departments were additionally reviewed. Exposures were grouped into categories manually for patient-level data and using a large language model (Claude, Anthropic) for ward-level supply data. After plausibility checks, these categories were used to identify product types that were common across cases (Figure 3). Categories routinely documented on individual patient-level (e.g. adrenalin, antibiotics, opioids) were not considered further in the ward-level analysis.

**Figure 3:**
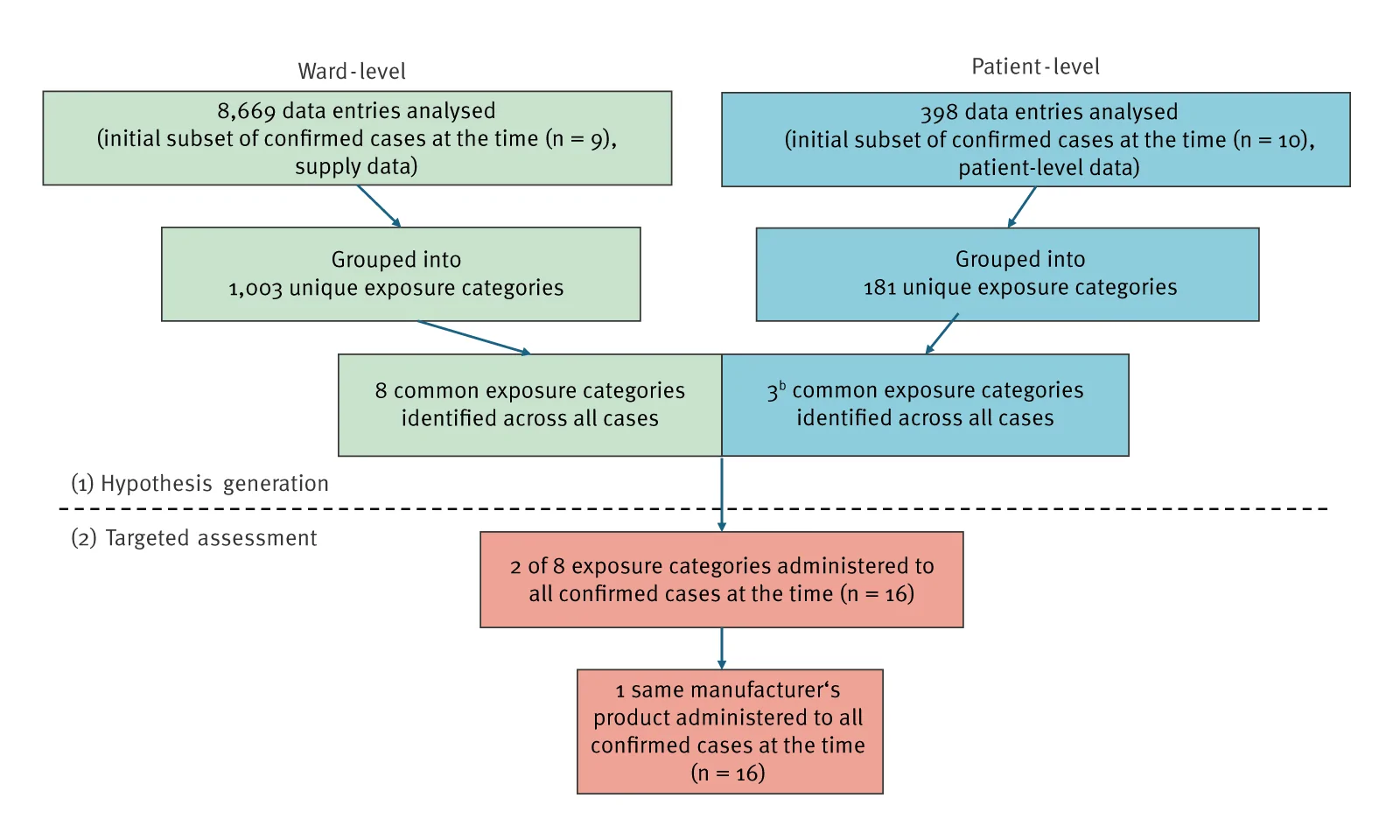
Flowchart of exposure data analysis, *Ralstonia mannitolilytica* outbreak, Germany, March 2026–September 2026 (n = 16 confirmed cases)^a^

^a^ Data up to 30 September 2026.

^b^ Of the three common exposure categories identified through patient-level analysis, two were documented and one was reported. All three were included among the eight common exposure categories identified through ward-level analysis.

### Hypothesis generation

Across patient-level data from 10 confirmed cases across four hospitals (Hospitals 1–4) we found two documented common categories among all cases: sodium chloride, and balanced electrolyte solution (Figure 3). In addition, prefilled flush syringes were not consistently documented in patient files, but all hospitals confirmed that such syringes had been used in all cases. We analysed ward-level data from nine confirmed cases at the time across three hospitals (Hospitals 1–3). Product category-based matching identified eight shared ward-level intravenous categories common on all wards of cases: prefilled flush syringe, sodium chloride solution, balanced electrolyte solution, water for injection, glucose, metoclopramide, dimenhydrinate, metamizole. Hospitals replied that according to documentation rules metamizole, metoclopramide and dimenhydrinate must routinely be documented on patient-level.

### Targeted assessment of suspected exposures

For a targeted assessment of those eight ward-level suspected exposures, we performed follow-up of 16 confirmed cases across seven hospitals (Hospitals 1–7), using a questionnaire to verify whether the suspected categories had been administered to cases and to obtain exact product names and manufacturers. This allowed us to distinguish exposures that were the same on the product-level from those that were only similar and matched solely on category-level (Figure 3). The survey showed that sodium chloride solution and prefilled flush syringes had been used in all cases, whereas the remaining six categories were only applicable to a subset of cases.

For sodium chloride solution, different products from different manufacturers were reported across cases. In contrast, for prefilled flush syringes, all cases were reported to have received flush syringes marketed under one trade name (see field safety corrective action (FSCA): https://www.bfarm.de/SharedDocs/Kundeninfos/EN/07/2026/30010-26_kundeninfo_en.html?nn=708434).

Lot numbers and corresponding article numbers of the suspected product were collected, determining that a single product had been in use on all wards with cases and likely administered to all confirmed cases. The importer provided a distribution list containing 218 hospitals in Germany that had received this product (of in total 1,841 hospitals in Germany [3]). All hospitals with cases were documented on the distribution list, except one hospital receiving medical products via a hospital on the list. No cases were reported by any of the 1,623 hospitals that had not received the product.

## Microbiological testing of suspected products

Given evidence from published studies that analytical methodology can substantially influence the likelihood of detection of *R. mannitolilytica* [4], a testing protocol was developed in close collaboration with the National Consiliary Laboratory for Cystic Fibrosis Bacteriology and the participating hospitals to optimise sensitivity. This testing protocol is further described in Supplement S1.

Targeted sampling and microbiological testing of prefilled flush syringes were conducted at the Robert Koch Institute, several hospital hygiene departments, and at the National Consiliary Laboratory for Cystic Fibrosis Bacteriology. All tests yielded negative results so far, but the work is ongoing.

## Implemented measures

Following notification from BfArM, the manufacturer agreed on a voluntary European Union (EU)-wide field safety corrective action including a temporary quarantine and a suspension of use on 3 July 2026: https://www.bfarm.de/SharedDocs/Kundeninfos/EN/07/2026/30010-26_kundeninfo_en.html?nn=708434. No additional cases have been detected since.

## Discussion

*Ralstonia mannitolilytica* is a non-fermentative, Gram-negative environmental bacterium commonly found in water and soil. Although rarely isolated from clinical specimens, it can cause severe infections such as bloodstream infections, particularly in immunocompromised patients [5,6]. Frequent antimicrobial resistance complicates treatment [7]. Due to its ability to survive in water and saline solutions, form biofilms, and pass through 0.2 µm sterilising filters, *Ralstonia* spp. can contaminate water systems, industrial production processes, and solution-based medicines or medical products [5,8,9]. Detection of the pathogen may be difficult using routine diagnostic methods [4]. In recent years, several nosocomial *R. mannitolilytica* outbreaks have been reported worldwide [4,10-13].

We identified 16 confirmed cases of *R. mannitolilytica* bloodstream infection across seven healthcare facilities in Germany. Epidemiological investigation identified the probable outbreak source in a specific product of prefilled flush syringes. As consequence of these results the manufacturer paused the distribution of the product and required customers to stop use of the products. Given that the product was also distributed to other European countries, potentially under different names, cases may occur elsewhere. Infections with *R. mannitolilytica* are not under routine surveillance and thus unlikely to be notified in many countries, and single cases detected in different locations may not be recognised as belonging to one outbreak. Consequently, timely communication and information sharing between healthcare institutions and public health authorities are essential to identify dispersed outbreaks.

Although robust epidemiological evidence linked the identified exposure to the outbreak, *R. mannitolilytica* could not be detected in the samples tested so far. Nevertheless, this does not exclude the product as the source of the outbreak. As *R. mannitolilytica* is particularly challenging to detect, we have defined a testing protocol to improve sensitivity. However, contamination may have been limited to specific syringes within a batch or occurred at very low bacterial levels.

As a limitation of our study, differences in procurement, storage, and documentation practices, and completeness of available data across hospitals may have affected the identification of shared exposures. An additional common product responsible for the outbreak may not fully be excluded. Furthermore, the prioritisation of identified exposures was informed by expert judgement and plausibility considerations.

## Conclusion

We describe a nosocomial outbreak of *R. mannitolilytica* infections affecting patients of various ages across different hospitals throughout Germany, epidemiologically linked to prefilled saline flush syringes. Rapid outbreak detection and case finding were facilitated by prompt information sharing of two affected hospitals and the detection of *R. mannitolilytica* infections in ARS, highlighting the importance of timely information sharing between hospitals and public health authorities. Although the distribution of the implicated product was paused and use of products was stopped, products have been distributed to other European countries. Potentially associated cases and new and recent isolates of *R. mannitolilytica* should be assessed for genomic relatedness to the German outbreak strain.

## Data Availability

Raw sequencing data have been submitted to European Nucleotide Archive (ENA) under BioProject ID PRJEB127983.

## Supplementary Data

211000 Supplement S1
