# Supplementary material for: Nosocomial outbreak of Ralstonia mannitolilytica associated with prefilled saline flush syringes distributed in Europe, Germany, March to September 2026
Source: Euro Surveill. 2026 Oct 8;31(40):2600819. doi: 10.2807/1560-7917.ES.2026.31.40.2600819 (PMC13653210; doi:10.2807/1560-7917.ES.2026.31.40.2600819)
Supplement: Supplementary file 1 [file 26-00819_BURDI_SupplementS1.pdf]

This supplementary material is hosted by *Eurosurveillance* as supporting information alongside the article "**Nosocomial outbreak *of Ralstonia mannitolilytica* associated with prefilled saline flush syringes distributed in Europe, Germany, March to September 2026**", on behalf of the authors, who remain responsible for the accuracy and appropriateness of the content. The same standards for ethics, copyright, attributions and permissions as for the article apply. Supplements are not edited by *Eurosurveillance* and the journal is not responsible for the maintenance of any links or email addresses provided therein.

Supplement S1

Protocol: Cultural detection of *Ralstonia mannitolilytica* from potential sources of exposure

1. Sample material & preparation

- Test samples typically contain low bacterial counts → enrichment by centrifugation or, in the case of large-volume containers, filtration is recommended – provided this is feasible in the laboratory

2. Culture media & inoculation

Option	Assessment
**Aerobic blood culture bottles**	Suitability checked, enrichment culture, usually automated shaking, sample volume up to 8–10 ml
**BHI liquid medium**	Suitability checked, enrichment culture, adequate oxygen supply required (automated/manual shaking)
**Other solid media**	Suitability checked, columbia blood agar, MacConkey agar

Important: Ensure an appropriate ratio of sample to culture medium

Note on FOS reagent: In exploratory testing, no difference was observed between blood culture bottles with and without the addition of supplements such as FOS (fastidious organism supplement) reagent – the addition of FOS reagent is not recommended.

3. Incubation conditions

Parameter	Recommendation
**Temperature**	36±1 °C is suitable; optimum growth at 32 °C,
**Oxygen**	Aerobic; ensure sufficient oxygen supply (accelerated growth)
**Incubation period**	5 days (extended incubation may be required if shaking or regular swirling is not possible)
**Swirling**	Usually automatic for blood culture bottles; for BHI, ensure this is done manually if necessary

4. Recommended procedure (summary)

1. Centrifuge the sample → Resuspend the sediment in a suitable volume
2. Aerobic blood culture bottles or BHI medium (ensure adequate oxygen supply)
3. Incubate at 36±1 °C for at least 5 days
